# How Different Quality Paradigms Undermine a Shared Value Base for Integrated Care: The Need for Collective Reflexivity

**DOI:** 10.5334/ijic.5935

**Published:** 2022-01-21

**Authors:** Everard van Kemenade, Marlou de Kuiper, Marjolijn Booij, Mirella Minkman

**Affiliations:** 1 Utrecht University of Applied Sciences, ACT, NL; 2 Utrecht University of Applied Sciences, NL; 3 University of Applied Sciences VIAA, NL; 4 Vilans, The Netherlands, NL; 5 TIAS School for Business and Society (TiU), NL

**Keywords:** quality paradigms, complexity, collective reflexivity, integrated care

## Abstract

In the development process of integrated care many impeding factors occur. Our premise is, that many of these barriers are related to the differences in values or perspectives. This article aims to clarify what an important challenge is for the further development of integrated care and for integrated care research. Professionals and managers in integrated care need to cope with and embrace uncertainty. However, that requires collective reflexivity. Collective reflexivity is a means to investigate the values of the partners interacting to co-create integrated care and to remove the roadblocks on the way.

## Introduction

In the development process of integrated care many impeding factors occur [1,2,3].

Researchers have found barriers that are general to all large-scale (inter-)organizational change, such as leadership and organizational culture. In Auschra’s systematic literature review of forty studies [4], for example, she mentions four main barriers: different professional backgrounds; organizational versus collective interests; lack of leadership and coordination; and lack of organizational resources and external funding. Our premise in this article is that many of these barriers to integrated care are related to the differences in values or paradigms. Shared norms and values have always been identified as factors in the success and failure of integrated care initiatives [5,6]. Integrated care requires new multidimensional governance mechanisms that connect organizations, sectors and people. Values play an important role in this, since (inter)organizational behavior, interaction and decision-making in integrated health services networks are strongly influenced by the values of the stakeholders involved [7]. However, those values lie hidden underneath these processes and are not often made explicit [8]. Following Zonneveld et al. [8, p. 2] values are defined as “meaningful beliefs, principles or standards of behavior, referring to desirable goals that motivate action”. Minkman [9] found 89 elements described as activities that seem relevant in multiple and very different integrated care settings. These activities support integration of processes and collaborative networks, but only partly address components of normative integration. To research the role of this normative integration, Zonneveld et al. [6] identified 18 values which play a role in integrated care practices. These values can be used as “a basis for the guidance of collaboration and governance processes in integrated care and add to conceptual knowledge and theory building of integrated care” (6, p. 8). This value related thinking is not absolute. It contributes to better understanding of the context of integrated care, behaviors of actors and its pluriformity. Furthermore, it is important to realise that besides that, values play a role in activities and integration of processes. Also, the quality paradigms that are used to reflect on integrated care developments are influenced by values themselves.

In this article, we aim to explore and clarify the challenge of values in the development of integrated care. We do so along two concepts: quality paradigms and collective reflexivity.

## Quality paradigms

Integrated care has often been thought of as an approach to improve the outcomes and quality of health and care services, but quality itself is a subjective term that depends on the viewpoint and preferences that different people place on what constitutes ‘quality’. Hence, any integrated care initiative will be significantly impacted by people’s interpretation of quality and what aspects of quality they ‘value’ over others. To clarify differences in the perspectives of quality management Van Kemenade and Hardjono [10] discerned four quality paradigms that also help to understand the differences between participants in the development of Integrated Care. The four paradigms are the empirical paradigm, the reference paradigm, the reflective paradigm and the emergence paradigm (see ***Table 1***).

**Table 1:** The four quality paradigms.


The **Empirical paradigm** likes to measure, develop measuring instruments, key performance indicators or to develop standards. Its values are accountability and accuracy. In research it embraces positivism as scientific philosophy. The **Reference paradigm** likes to develop models, protocols, pathways to show how to achieve quality. Its values are success and improvement. It embraces constructivism as scientific philosophy. The **Reflective paradigm** has the adage that quality cannot be defined, just discussed. The reflective paradigm likes to reflect professionally and between professionals on what good quality is. In the quality event people, their interactions, conceptions and worldviews are the objects of the study, and these are reflected upon. In this paradigm the research method is often the expert opinion, viewpoint, or the perspective of a (group of) researcher(s) on a theory or topic. Its values are wisdom and professionalism. It embraces subjectivism as scientific philosophy. The **Emergence Paradigm** states that radical innovation emerges out of the interaction and co-creation of a diverse group of people, including professionals, patients, and experts by experience. Its values are willingness to change and flexibility. It embraces complexity theory as scientific philosophy.

**The case of Vince**

Vince is a 17-year-old boy. At this moment he is on remand, because he is suspected of attempted manslaughter during the riots that occurred as a reaction to the COVID-curfew. What happened before? Vince was born in 2004 in a family consisting of a father, mother and half-brother, four years younger. His father soon left, what made his mother to work hard to feed the two boys. Vince has no contact with his father anymore since. He had difficulties at school. Because of his behavioral and learning problems he had to switch from his familiar high school to a school for secondary special education. The social team in the neighborhood tried to support Vince and his family. Vince was diagnosed with ADHD. By now he has a long history of youth care programs, including an admission to a French clinic for youth with severe behavioral problems.

When we analyse the case of Vince here, we see facts and figures. We see the empirical paradigm.

At the age of 15 Vince made it to a quite promising bass guitar player, until he broke his wrist in a football game. His chances to achieve his dream of becoming a successful musician have gone. What he could do so well, vanished in a flash. There he was, without a school diploma because of the admission in the French clinic. He did not know, why he needed a diploma after all. The attendance officer searches with Vince for a possible career that would fit him. Slowly, Vince gets motivated to become a pastry chef. He did several intakes in bakery schools. One intake did not take place, because the school made a double appointment; another school acts tough because of his past in youth care; another considers him not admissible because lack of diploma. Vince gets demotivated and does not show up at the meetings with the attendance officer anymore.

When we analyse the case of Vince here, we see professional reflection on what happened. We see merely the reflective paradigm.

Next week Vince will become 18. He knows that he will have to pay his own bills. His mother can no longer support him. He does not know where to start. How can I find a job so fast? The stress rises and his self-confidence continues to decline. The attendance officer advises him to ask for an allowance paid by the municipality. The counselor at the municipality asks him difficult questions and explains that he must search for a job for four weeks, before he can qualify for a pay. She sees that Vince is breaking down and suggests arranging guidance for Vince in getting his life on track. Vince leaves upset, full of helplessness and frustration. But, Vince knows, he needs to do something and cooperate with them. He wants to talk with an expert by experience.

When we analyse the case of Vince here, we see that out of the interaction between Vince and the counselors an insight occurs. We see the emergence paradigm.

Soon Vince will come out of remand. There is not enough proof that he was seriously involved in the curfew riots, but he gets a fine for violating the curfew. Vince is happy with that, wants to get into action, but still does not know what to do.

His coach from the municipality has heard Vince is coming home and sends him a WhatsApp. She tells him, she has a plan. She has a list of employers that want to give young people a chance, regardless of their past. She has discussed with her colleagues that he can get money from the municipality until he has a job or an education. The coach offers Vince to do a career choice test. Maybe there is another education that relates to his love for music.

Vince sees light at the end of the tunnel again. He cannot wait until he gets and tell his mother about his future plans.

When we analyse the case of Vince here, we see that after the emergence of Vince’s willingness to act, the coach was there to provide a plan, at the same time creating a model for social workers to behave in cases like this. We see the reference paradigm.

To illustrate how the paradigms can play a role in integrated care reality, we introduce the case of Vince.

**Figure 1:**
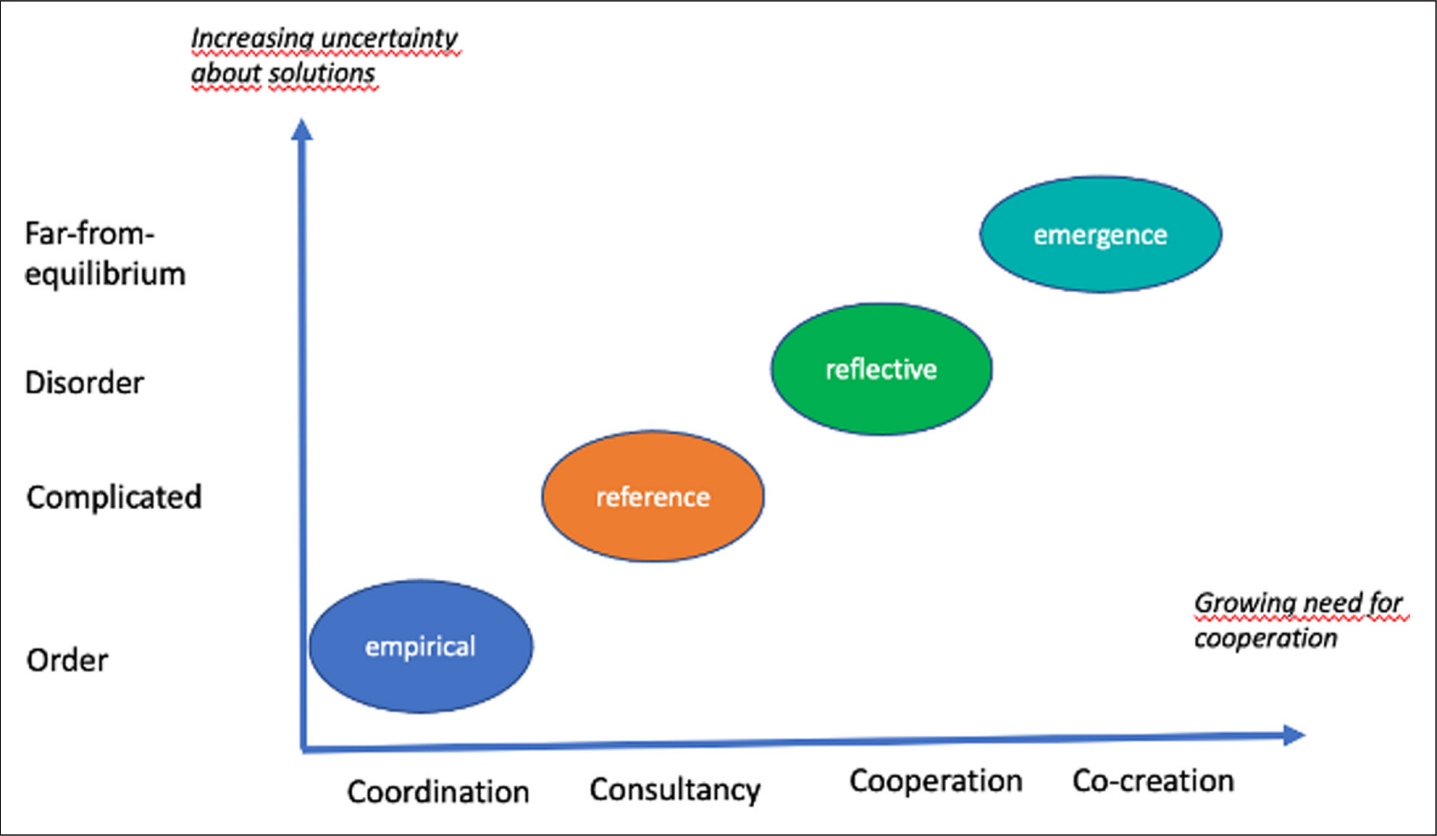
Paradigms and context.

Besides the fact that we have four different quality paradigms that people in integrated care programs may have a different preference for (i.e. a different value-base), the balance of these preferences strongly changes according to the context of the integrated care program. The empirical paradigm fits best in a context of order or equilibrium, in a situation where we are relatively certain about the solutions of the problems at hand. The reference paradigm fits best in a complicated context. In such circumstances one can measure or make plans to check and act upon (following the PDCA-cycle). When the uncertainty about solutions to the problem and complexity are increasing, the reflective (disorder) and the emergence paradigm (far-from-equilibrium) could fit better, see ***Figure 1***.

### Complexity in integrated care research

In exploratory research on the paradigms in integrated care, Van Kemenade, Van der Vlegel and Van der Vlegel [11] analyzed 255 articles in the International Journal for Integrated Care to explore which paradigm in integrated care is currently dominant, if paradigms are lacking and from which perspectives and views on integrated care research is conducted.

**Figure 2:**
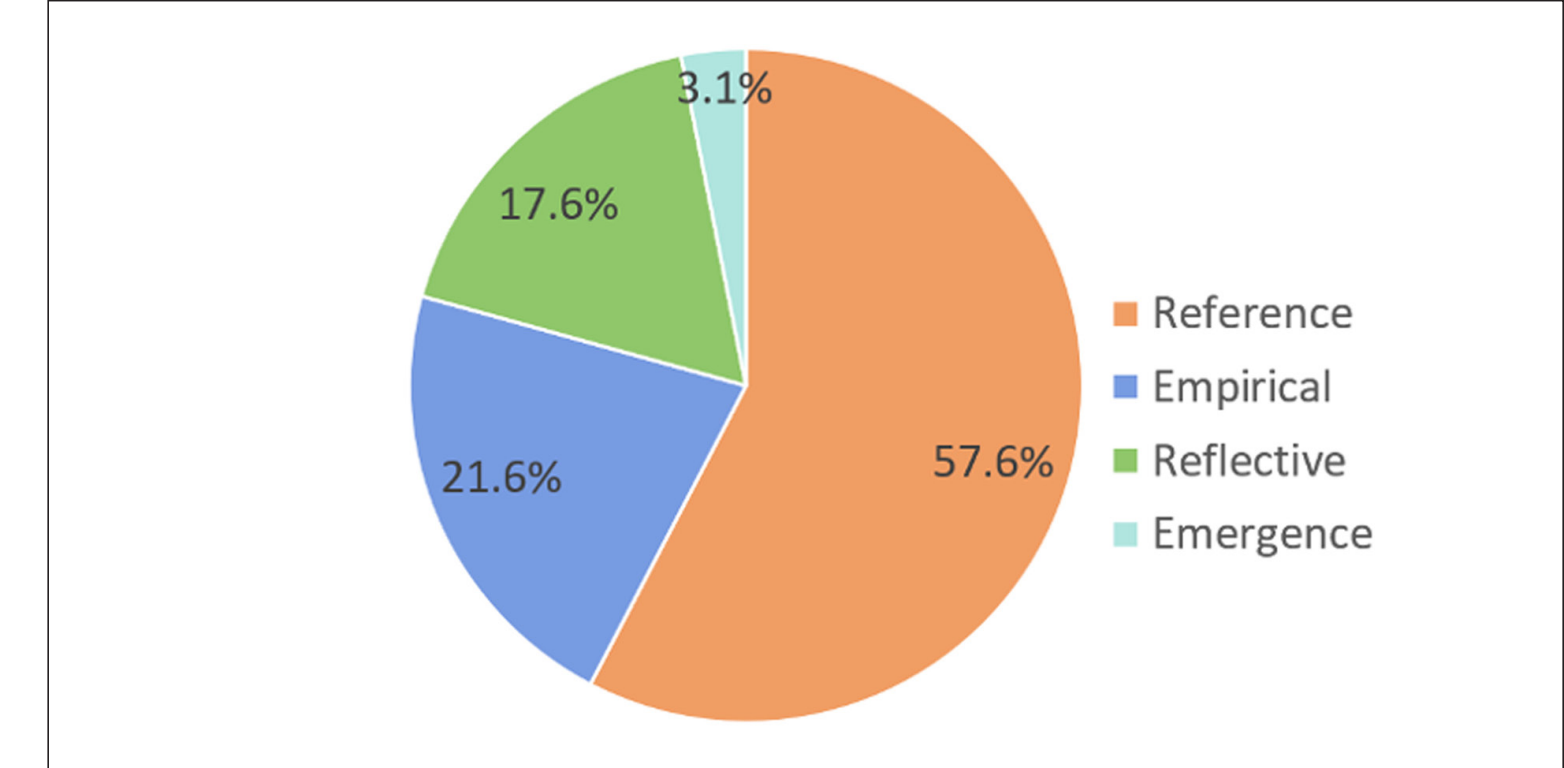
Percentage of articles in each paradigm (total n = 255).

Integrated care research in the last twenty years is found to be dominated by the reference paradigm (57,6%) and the empirical paradigm (21,6%). Only 46 articles (17,6%) represented the reflective paradigm. And only 3,1% of the articles was written from the emergence paradigm (see ***Figure 2***).

At this point we can conclude that the empirical and reference paradigms are dominant in integrated care research, that more value is based on these paradigms, while integrated care development might (also) require emergence and reflexivity, since these fit best in times of disorder and far-from-equilibrium situations, in uncertainty and complexity. For integrated care development, often a complex and uncertain mix of interventions, behaviors, professional perspectives, and timeframes are needed, for which can be questioned if appropriate measurements, models and protocols (as named in the empirical and reference paradigm) do occur. Further, preferences and values of involved researchers for certain paradigms can change over time or related to changing purposes or contexts. This mismatch of values and perspectives can contribute to the subsequent problems in the integrated care enterprise.

## Collective reflexivity

Given the unbalance in values and paradigms used, there is a need within the development of integrated care for a more balanced process to give voice to a wider or more appropriate set of values and perspectives. Starting from reflection and including the multidimensional values present in integrated care settings, we present reflexivity and collective reflexivity.

In the reflective paradigm the professionals *reflect-on-action*. Schön [12] also discerns *reflection on reflection*. That meta-level brings us to reflexivity. Reflexivity is proposed to be the ‘regulative ideal’ that links the different paradigms [13]. Reflexivity is defined by Lincoln & Guba [14] as the process of reflecting critically on the self as a researcher, the ‘human as instrument’. We share the vision that a reflexive questioning of values and normative orientations is not sufficiently acknowledged [15], also in integrated care.

Reflexivity is seen to be a collective process, that can lead to the transformation of a social system [13]. Reflection most of the time is individual, where reflexivity undoubtedly is relational [16]. West [17] defines team reflexivity as “the extent to which group members overtly reflect on, and communicate about the group’s objectives, strategies (decision making) and processes (communication) and adapt these to current or anticipated circumstances” [17, p.3]. Rieu describes an example during the disruption that the disaster of Fukushima was. Since the date of the 11 of March 2011 Japan developed a collective reflexivity by gathering narratives of witnesses, giving voice to casualties, investigating causes and responsibilities, debating on the consequences and answers to the disaster, by reconstructing their lives and innovating the institutional system that made the disaster possible [18]. This competence of reflexivity needs more attention, especially as a collective process.

We suggest collective reflexivity for making existing values more explicit, starting or facilitating the co-creation process. Or to conclude that value hierarchies differ too much, are not shared or cannot be negotiated towards a common goal.

## Conclusion

In this perspective paper, we present no firm solutions but aim to explore on what one of the challenges is for the further development of integrated care and for integrated care research. We did so along two concepts: quality paradigms and collective reflexivity.

Collaborative reflexivity about the differences especially in values and in paradigms used, can contribute to understanding and overcoming the barriers for development of integrated care and integrated care knowledge. Repeatedly the use of the four paradigms in the actual context needs to be discussed and the underlying values to be exchanged or even shared. Such a collective reflexivity requires knowing and applying complexity theory. It requires the principle of equivalency as well as accepting there might be inequality. Reflexivity could be an important competence for professionals in integrated care development, and for leaders and coordinators of involved organizations and networks. The role and merits of reflexivity seems to be undervalued and should be further explored. Using this meta-reflective approach could tow away (some of) the roadblocks for integrated care. A reflexive questioning of values, background assumptions and normative orientations needs to be acknowledged, also in the development of research frameworks. Not the outcome of that deliberation, but it is the co-creation that counts, as the case of Vince also proofs.
